# HIV: An Epidemiologic study on Head and Neck Involvement in 50 Patients

**Published:** 2014-04

**Authors:** Mehdi Bakhshaee, Mohammad Reza Sarvghad, Kamran Khazaeni, Rahman Movahed, Ali Mohammad Hoseinpour

**Affiliations:** 1 *Sinus and Surgical Endoscopic Research Center, School of Medicine, Mashhad University of Medical Sciences, Mashhad, Iran.*; 2 *Department of Infectious disease, School of medicine, Mashhad University of Medical Sciences, Mashhad, Iran.*; 3 *Department of Otorhinolaryngology-Head & Neck Surgery, School of Medicine, Mashhad University of Medical Sciences, Mashhad, Iran.*; 4 *General Practioner, Mashhad University of Medical Sciences, Mashhad, Iran.*

**Keywords:** AIDS, HIV, Head, Neck, Otologic, Oral, Rhinologic

## Abstract

**Introduction:**

Acquired immunodeficiency syndrome (AIDS) is a worldwide infection. Because of the vast array of manifestations of AIDS and its many atypical presentations, it is becoming increasingly challenging for clinicians to accurately diagnose new lesions.

**Materials and Methods:**

In a descriptive cross-sectional study conducted from 2007 to 2010, 50 patients with a proven human immunodeficiency virus (HIV) infection were evaluated. Based on the findings of a physical examination and paraclinic tests, HIV signs and symptoms were recorded.

**Results:**

The mean (range) age of the patients was 35.45 ±5.24 (5–55) years. Forty-two (84%) cases were male and eight were female. The mean duration of carrying the virus was 4.51 ±1.03 years. Oral manifestations were the most common (94%), followed by rhinologic (88%), otologic (66%), and finally neck (44%) manifestations.

**Conclusion:**

Head and neck presentations are very common in HIV patients; therefore otolaryngologists, as the first physicians who may encounter such patients, should be aware of this condition.

## Introduction

Human immunodeficiency virus (HIV) infection/ acquired immunodeficiency syndrome (AIDS) is a global pandemic. Today, it is estimated that 34 million people worldwide are infected by this virus, of which two-thirds live in the sub-Saharan regions of Africa; 50% are female and 2.5 million are under the age of 15 (1). AIDS is a fatal disease, which compromises the body's immune system and leaves the victim vulnerable to life-threatening opportunistic infections, neurologic disorders, or unusual malignancies (2). The key manifestation of HIV infection is a severe immunodeficiency state which is principally caused by diminished CD4 T-lymphocytes. This condition results in the high rate of opportunistic diseases, especially infections and neoplasms. 

Approximately 80% of patients with HIV infection initially present with otolaryngo- logical symptoms (2). Head and neck manifestations of AIDS can involve the skin, ear, upper aero-digestive tract, and neck (3), as well as almost all other organs. It is becoming increasingly challenging for clinicians to accurately diagnose new lesions, due to the vast array of manifestations of AIDS in this region and their many atypical presentations (2,4). Sinonasal symptoms are among the most common complaints in HIV-positive patients and the maxillary and ethmoid sinuses are frequently involved. Otologic manifestations are observed in 56% of cases. External otitis is common and different types of sensorineural hearing loss (SNHL) are seen in 49% of HIV patients. In addition, unilateral and bilateral facial nerve palsy is 100% more prevalent among such cases. Oral candidiasis is the most common feature of AIDS in the oral cavity, with a prevalence of 70–90%. Non-Hodgkin’s lymphoma and Kaposi sarcoma have a strong correlation with HIV infection and are described as the AIDS-defining conditions. Eventually, cystic enlargement of the parotid gland and Kaposi's sarcoma are increasingly being encountered in the head and neck examination of HIV-infected patients (2-4).

Given the importance of opportunistic pathogens and related cancers, with respect to the growing number of HIV cases in our country, and because very few studies have been conducted in our region to date; we decided to study the clinical signs and symptoms of this disease in the head and neck region. We also evaluated the prevalence of opportunistic infections and neoplasms at different stages of the disease and different levels of immunodeficiency. 

## Materials and Methods

In a descriptive cross-sectional study conducted from 2007 to 2010, 50 patients with HIV infection were evaluated. Patient records, available at the AIDS clinic at our health center, Mashhad, Iran, were initially studied and then the patients were asked to visit the clinic for a further evaluation. A thorough medical history was taken and a physical examination of the head and neck area was performed on each patient. The variables studied, including medical history, physical examination, demographic data and paraclinic laboratory tests, were reviewed. Based on the findings of a physical examination and paraclinic tests, patients with head and neck involvement were prescribed essential medication and repeated examination and follow-up visits were considered.The data obtained were analyzed using SPSS software, version 13 and applying appropriate statistical tests. 

## Results

Fifty cases were enrolled in this study, with a mean (range) age of 35.45 ±5.24 (5–55) years. Forty two (84%) cases were male and eight were female.

The TCD4 level was <200/µl, 200–500/µl, and >500/µl in 18.2%, 34.1%, and 47.7% of cases, respectively, and the mean CD4 T-cell count was 471/µl in males and 539/µl in females. The mean duration of carrying the virus was 4.51 ±1.03 yrs. Demographic and epidemiological characteristics of the studied patients are listed in (Table 1).

**Table1:** Epidemiological, clinical and demographic characteristics of the 50 patients with AIDS

		**Number**	**Percent**
Education	Uneducated	40	80
	Educated	10	20
Job	Busy	26	52
	Jobless	24	48
Marital Status	Married	19	38
	Single	9	18
	Divorced	12	24
Transmission Type	IV injection	38	76
	Sexual	11	22
	Maternal	1	2
Co-infections	HBS-Ag+	6	12
	HCV+	34	68
	VDRL or FTA_ABS+	11	22
T CD4 Cell Count	<200	9	18
	200-500	17	34
	>500	24	48

Otologic signs and symptoms were diagnosed in 67.3% of cases, with external otitis and tinnitus being the most common. Nasal signs and symptoms were observed in 89.7% of the patients, while rhinorrhea was the most common complaint. Oral cavity and the oropharynx were involved in 95.9% of cases, with dental carries being the most common finding. The prevalence of symptoms in the neck and salivary glands was 44.8%, and cervical adenopathy was the most common finding.

The frequency of otologic, rhinologic, oropharyngeal, and head and neck signs and symptoms are presented in Table 2.

**Table 2:** Symptoms and signs in different area of head and neck among 50 patients with AIDS

**Otologic**		**Rhinologic**		**Oral and Oropharyngeal**		**Head and Neck**	
Symptoms and signs	Percent	Symptoms and signs	Percent	Symptoms and signs	Percent	Symptoms and signs	Percent
Otalgia	10%	Rhinorrhea	50%	Mucosal Ulcer	6%	Neck Mass	2%
Otorrhea	16%	Congestion	28%	Xerostomia	26%	Cervical Adenopathy	38%
External Otitis	28%	Epistaxis	18%	Odynophagia	14%	Goiter	2%
Hearing Loss	24%	Sneezing	12%	Dysphagia	4%	Sialorrhea	2%
Tinnitus	28%	Snoring	20%	Post-nasal Discharge	32%		
Vertigo	18%			Dental Carries	44%		
Tympanic membrane perforation	14%			Herpetic Infection	2%		

## Discussion

The mean age of the participants in this study was approximately 35 years, demonstrating a trend for infection among our young population. The mean age of males was significantly higher than that of females (37 vs. 27 yrs). In contrast with other developing countries, male predilection was observed in our study, which is a characteristic of regions where the most common transmission route is not sexual contact. However, it is predicted that this proportion will change in the near future.

Eighty percent of the patients had a low level of education or were uneducated, and 40% were unemployed. Low educational status and unemployment puts individuals at risk of social problems and subsequently risky behaviors. It seems that social isolation and unemployment occurs secondary to HIV infection in our society.

Eighty-two percent of patients were married but at the time of study entry, just 40% lived with their spouse. In total, 76% (90% of males) had a history of imprisonment.

The mean CD4 T-cell count was 471/µl in males and 539/µl in females; which had not a meaningful difference. Among patients at the “AIDS” clinical stage, 87.5% were male while 12.5% were female; showing a significant difference.

The main route of transmission was shared intravenous (IV) needles (76% of cases; 92% among males). All females were infected through sexual contact. This pattern was suggestive of a period of males’ involvement via shared needles in prisons, and a subsequent second wave of infection through sexual contact.

Twelve patients had HBS-Ag in their sera, 68% were hepatitis C virus (HCV) positive, and 22% showed a positive venereal disease research laboratory (VDRL) test. All were seen in IV-transmitted patients and no female was infected by these pathogens.

Otologic involvement in HIV patients is less common in comparison with other manifestations.

In our study ear-related signs and symptoms were found in 66% of patients and were not related to the CD4 T-lymphocytes count.

In the literature, ear complaints have been reported in up to 56% of HIV cases (5). Hearing loss, otalgia, and otorrhea are the most common manifesting symptoms in some studies (6), while sensorineural hearing loss, external otitis, acute otitis media, and serous otitis media are the most common otologic disorders in others (7). In a case series by Chandrasekhar et al. otologic manifestations were reported in 33% of HIV patients (8). A total of 34% of his studied patients had fullness of the ear, 23% showed otalgia, 26% had tinnitus, 32% suffered from dizziness, 29% had hypoacusis, and 5% had otorrhea (8). In a long-term study by Prasad et al, 968 HIV patients were observed over the course of 8 years, and otologic involvement was observed in 20% with chronic otitis media being the most common finding, with 13% prevalence (9).

In a similar study by Jafari et al in Iran, hearing loss (61%), post-nasal discharge (23.5%), xerostomia (39.8%), and voice change (23.5%) were the most frequently cited complaints by participants whereas external otitis (6%) was found to be the most common sign (10).

In the present study, 16% of the cases complained of otorrhea, 28% of tinnitus (the rate of tinnitus in the normal population appears to be 3–30%), 24% of hearing loss, and 28% of ear canal pruritus. Eleven cases (22%) had COM (Chronic Otitis Media), as proven by examination, history taking, and imaging. Bernaldez et al. reported the prevalence of COM as 13.24% in seropositive children over 10 years, with a 3.31% rate of new cases each year (11).

One of our patients had multiple perforations in the tympanic membrane, ultimately diagnosed as middle ear tuberculosis, and underwent medical therapy. Sensorineural hearing loss was seen in just one of the 12 patients with hearing loss (2%), most probably due to interferon use which has been reported to cause only transient hearing loss (12). In the literature, this type of hearing loss was reported in 23–49% of HIV patients, with anticipated causes including central nervous system (CNS) or vestibule- cochlear nerve involvement with HIV; although ototoxic drugs and neoplasms should not be overlooked. Forty-two percent of cases with otologic manifestations had a CD4 level over 500/µl and 18% had a CD4 level below 200/µl.

Sinonasal symptoms are among the most common complaints in HIV-positive patients. Around 80% of HIV-positive cases experience an acute sinusitis episode and 58% have either acute or chronic sinusitis at the time of referral (13–15). Generally, the prevalence of rhinosinusitis ranges from 30–68% in HIV patients (16). Yet, in a 6-month period, the rate of sinonasal symptoms was similar among HIV cases and the general population (17). Moreover, the responsible microorganisms are relatively similar to those of the general population, with the exception of higher Staphylococcal and Pseudomonas infections (9,18). Eighty-eight percent of our patients had signs and symptoms of sinonasal disorders and there was no relation between the clinical stage of the disease and these manifestations; although one should consider opportunistic and uncommon pathogens when the CD4 T-lymphocyte count is below 200/µl (18). Fifty percent of patients reported rhinorrhea (mostly clear) while it was diagnosed in only 32% cases in nasal examination. Nasal congestion, snoring, recurrent epistaxis, septal deviation, and repetitive sneezes were found in 28%, 20%, 12%, 64%, and 12% of patients, respectively. Miziara et al. reported nasal congestion, purulent rhinorrhea and headache as the most common complaints in HIV patients with chronic rhinosinusitis (19). While 10.2% of our studied patients showed signs and symptoms of allergic rhinitis, the rate of allergy in HIV patients is reported to be twice the normal population (18). In addition, 44% of the cases with sinonasal manifestations had CD4 levels over 500/µl, whereas it was below 200/µl in 20%.

In the oral cavity, candidiasis, Kaposi sarcoma, hairy leukoplakia, gingival and periodontal infections, aphthous, herpes simplex stomatitis and xerostomia are the most common manifestations (12). Candidiasis is the most common oral symptom in HIV patients and its prevalence is reported as up to 90% in the AIDS clinical stage (12,20); whereas hairy leukoplakia is considered pathognomonic for HIV (14,15). HSV infection of the oral cavity is seen in nearly all HIV clinical levels and Herpes Labialis is the most common manifestation (21,22). A total of 94% of our patients showed signs and symptoms of oral cavity involvement. In total 6% had oral mucosal lesions, xerostomia was reported in 26% while its prevalence has been previously reported as 7–14% (23,24). This may justify the high rate of dental and periodontal disorders in our study. Gingival erythema was found in 10% of individuals, while its prevalence is 2–6% in developing countries (25). Although several oral cavity manifestations are known to be related to CD4 levels (20,26–29), in our study no significant correlation was found. This may be due to the small study population. A total of 48% of the cases with oral cavity manifestations had CD4 levels over 500/µl whereas 19.0% had CD4 levels below 200/µl.

An enlarged cervical lymph node could be considered as the most frequent finding in seropositive patients (9). Reactive follicular hyperplasia is the most common etiology for cervical lymphadenopathy in this population and occurs in 12–45% of such cases (30,31).

Parotid glands are involved in 5–10% of HIV patients (32) which could be the result of benign or malignant processes. A benign lymphoepithelial cyst (BLC) (is a benign characteristic pathology of the parotid glands which is probably the result of ductal metaplasia secondary to intra-parotid lymph node enlargement. It is almost always seen in the initial phase of the disease and is accompanied by generalized lymphadenopathy (3,4,13,33). Forty-four percent of our patients had signs and symptoms of neck and salivary gland disorders. Persistant generalized lymphadenopathy was found in 38% of patients. In general, 48% of cases with neck and salivary gland manifestations had CD4 levels over 500/µl while 26% had CD4 levels below 200/µl.

## Conclusion

The otorhinolaryngologic and head and neck manifestations of AIDS are common and widespread. Therefore, otorhinolaryn- gologists should be aware of this condition in order to perform a more effective evaluation and consider the most appropriate treatment for this group of patients.

## Financial Disclosure:

No financial disclosures
